# Melanin-dependent tissue interactions induced by a 755-nm picosecond-domain laser: complementary visualization by optical imaging and histology

**DOI:** 10.1007/s10103-023-03811-4

**Published:** 2023-07-14

**Authors:** Kevin Jacobsen, Vinzent Kevin Ortner, Gabriella Louise Fredman, Rikke Louise Christensen, Christine Dierickx, Emil Tanghetti, Uwe Paasch, Merete Haedersdal

**Affiliations:** 1 Department of Dermatology and Wound Healing Centre, Copenhagen University Hospital, Bispebjerg and Frederiksberg, Nielsine Nielsens Vej 17, Entrance 9, 2Nd Floor, NV DK-2400 Copenhagen, Denmark; 2 Skinperium, Private Dermatology Clinic, Rue Charles Martel 52, 2134 Luxembourg, Luxembourg; 3 Center for Dermatology and Laser Surgery, 5601 J Street, Sacramento, CA USA; 4 Department of Dermatology, Venereology and Allergy, University of Leipzig, 04103 Leipzig, Germany

**Keywords:** Picosecond-domain laser, Optical coherence tomography, Line-field confocal optical coherence tomography, Ex vivo reflectance confocal microscopy, Melanin, Lasertissue interaction, Laser-induced optical breakdown, Histology Digital staining

## Abstract

Fractional picosecond-domain lasers (PSL) induce optical breakdown, which correlates histologically to vacuolization in the epidermis and dermis. In this ex vivo porcine study, we sought to establish a framework for the investigation of laser-tissue interactions and their dependence on melanin density. Light- (melanin index: 24.5 [0–100]), medium- (58.7), and dark-pigmented (> 98) porcine skin samples were exposed to a 755-nm fractional PSL and examined with dermoscopy, line-field confocal optical coherence tomography (LC-OCT), conventional OCT, and subsequently biopsied for digitally stained ex vivo confocal microscopy (EVCM) and histology, using hematoxylin and eosin (HE) and Warthin-Starry (WS) melanin staining. Dermoscopy showed focal whitening in medium- and dark-pigmented skin. Similarly, LC-OCT and OCT visualized melanin-dependent differences in PSL-induced tissue alterations. Vacuoles were located superficially in the epidermis in dark-pigmented skin but at or below the dermal–epidermal junction in medium-pigmented skin; in light-pigmented skin, no vacuoles were observed. Histology confirmed the presence of vacuoles surrounded by areas void of WS staining and disrupted stratum corneum in darker skin. The combined use of optical imaging for multiplanar visualization and histological techniques for examination of all skin layers may mitigate the effect of common artifacts and attain a nuanced understanding of melanin-dependent laser-tissue interactions.

**Supplementary Information:**

The online version contains supplementary material available at 10.1007/s10103-023-03811-4.

## Introduction

Since their FDA approval in 2012, picosecond-domain lasers (PSL) have been under steady development. After the approval of Alexandrite crystal for 755-nm lasers, other PSL media such as neodymium-doped yttrium aluminum garnet (Nd:YAG) crystal for 532-nm or 1064-nm wavelengths soon followed. PSL can deliver energy at pulse durations in the 300–900 ps range, causing photothermal and photomechanical tissue interactions [1–3].

Using a diffractive lens array, fractionated PSL devices can generate focused beams of higher peak energy that lead to chromophore-assisted ionized plasma formation, a phenomenon commonly referred to as laser-induced optical breakdown (LIOB). LIOB can occur both in artificially and naturally pigmented skin and corresponds histologically to the immediate appearance of intraepidermal vacuoles [1, 4].

While initially introduced as a faster and more tolerable method for tattoo removal, PSL have since undergone a broadening of their clinical indications, including pigmentary disorders, scar revision, and photorejuvenation [1]. Recently, the use of PSL has also been investigated in the context of laser-assisted drug delivery [5], a technique that conventionally relies on fractional ablation of the skin’s surface to improve cutaneous delivery [6].

To advance the preclinical development and clinical implementation of PSL devices, a complete picture of the entire spatiotemporal spectrum of PSL-induced morphological changes is warranted. Previous investigations of 755-nm PSL-tissue interactions (see Suppl. Table 1) used conventional HE histology to visualize and assess LIOB. However, identification of PSL-tissue interactions is limited by well-known tissue processing artifacts that can mimic the appearance of vacuoles [7]. 

Optical imaging devices, such as reflectance confocal microscopy (RCM) and optical coherence tomography (OCT), are widely used in dermatological research to evaluate laser-tissue interactions noninvasively [8–11]. A previous investigation by Tanghetti et al. successfully incorporated RCM to visualize the development and migratory pattern of epidermal vacuoles following exposure to a 755-nm PSL; [12] similarly, Hwang et al. used high-resolution OCT imaging of 1064-nm PSL-treated areas to describe the immediate formation of vacuoles and the gradual accumulation of cellular debris [13].

Emerging technologies such as line-field confocal optical coherence tomography (LC-OCT) and bimodal ex vivo confocal microscopy (EVCM) with cellular resolution have been employed in preclinical settings to facilitate the identification and evaluation of energy-based device-induced tissue effects [12, 14–19]. However, their utility in the visualization of PSL-tissue interactions remains to be investigated.

In this exploratory investigation, we sought to gain a deeper understanding of PSL-tissue interactions by using an integrative approach of optical imaging techniques and histological stains to characterize and compare the micromorphology and melanin dependence of PSL-tissue interactions.

## Materials and methods

### Study design

An observational descriptive ex vivo study was carried out at the department of dermatology at the Copenhagen University Hospital, Bispebjerg, Denmark. Porcine skin with three different melanin densities were treated with a PSL and subsequently assessed by optical imaging and histological examination. Table 1 presents an overview of the number of optical images and histological sections acquired for each PSL-treated group.

**Table 1:** Overview of data collection for dark-, medium-, and light-pigmented ex vivo porcine skin

Melanin density	Line-field confocal optical coherence tomography (LC-OCT)	Optical coherence tomography (OCT)	Bimodal ex vivo confocal microscopy (EVCM)	Hematoxylin–eosin and Warthin-Starry histology
Dark	4 treatment areas	6 treatment areas	6 treatment areas	6 treatment areas
	*n* = 2 3D images per area	*n* = 6 images	*n* = 6 biopsies	*n* = 6 biopsies
	*n* = 8 3D images		*n* = 6 VivaBlock images	*n* = 30 images
Medium	4 treatment areas	6 treatment areas	6 treatment areas	6 treatment areas
	*n* = 2 3D images per area	*n* = 6 images	*n* = 6 biopsies	*n* = 6 biopsies
	*n* = 8 3D images		*n* = 6 VivaBlock images	*n* = 30 images
Light	4 treatment areas	6 treatment areas	6 treatment areas	6 treatment areas
	*n* = 2 3D images per area	*n* = 6 images	*n* = 6 biopsies	*n* = 6 biopsies
	*n* = 8 3D images		*n* = 6 VivaBlock images	*n* = 30 images
Total	*n* = 24 LC-OCT 3D images	*n* = 18 OCT images	*n* = 18 VivaBlock images	n = 90 digital histology images

### Skin samples and laser treatment

We harvested three types of full-thickness skin from the backs of Duroc crossbreeds (Duroc × Danish Landrace × Yorkshire) and Piebald Danish Landrace. These porcine skin types resemble human skin in their histological architecture and cover a large spectrum of melanin density. Before treatment, we cleansed samples for subcutaneous tissue, shaved all hair, and noninvasively measured skin reflectance to determine its melanin density (Skintel® Melanin Reader™, Palomar Cynosure) and calculate the average melanin index (MI) for each skin type (MI; 0–100) [20, 21].

Samples were stored at − 80 °C and thawed to room temperature before processing with a picosecond-domain 755-nm Alexandrite laser (PicoSure® with FOCUS focused lens array, Cynosure, Westford, MA, USA) with a fractional optic. Each sample was exposed to three single-shot passes with a 6-mm spot size at a fluence 0.71 J/cm^2^.

### Optical imaging

We used three different commercially available optical imaging modalities: two in vivo devices (optical coherence tomography (OCT), line-field confocal OCT (LC-OCT), and one ex vivo imaging system (ex vivo confocal microscopy (EVCM)). For co-localization between in vivo techniques, a customized adhesive template (Leukoplast® Fixomull® transparent, Germany) was placed on top of PSL-exposed areas. Images were evaluated qualitatively in ImageJ (1.49) and proprietary software suites (Vivosight, DAMAE, MAVIG).

#### Line-field confocal optical coherence tomography (LC-OCT)

The LC-OCT device (DeepLive™, Damae, Paris) combines the concepts of time-domain OCT and confocal microscopy. It comes equipped with a supercontinuum laser (centered at 800 nm) as a broadband light source and a high numerical microscopic objective (0.5 NA), thus providing cellular images with an isotropic resolution close to 1 µm. After examination of the PSL-processed skin with the integrated dermoscopic camera (i.e., dermoscopy) of the LC-OCT device, mineral oil as an immersion liquid was applied before acquisition of three-dimensional images over a 1200 × 500 × 500 µm^3^.

#### Optical coherence tomography (OCT)

Using a Multi-Beam Swept-source Frequency Domain OCT device (Vivosight® Dx, Michelson Diagnostics, Kent, UK) equipped with a 1035-nm laser, we acquired 6 × 6 mm structural OCT scans consisting of 250 cross-sectional images with a resolution of close to < 7.5 µm laterally and < 5 µm axially that were subsequently reconstructed into en face images.

#### Ex vivo confocal microscopy (EVCM)

We used a bimodal EVCM system (Vivascope 2500 M-G4, Mavig©, Germany) for combined reflectance (785 nm) and fluorescence (488 nm) imaging of 8-mm punch biopsies (Biopsy Punch KAI, PFM Medical, Germany) at a resolution of < 1.25 µm and < 5 µm in axial and lateral direction, respectively. For EVCM, biopsies of PSL-treated area were bisected, rinsed in distilled water, and submerged for 30 s in an Acridine Orange solution (Exλ = 460–500 nm; Emλ = 526–650 nm), a fluorescent staining agent to enhance nuclear contrast.

After a second rinse, the biopsies were transferred to a glass slide and flattened using a magnetic tissue compression device [22]. We captured a mosaic of stitched confocal microscopy grayscale images (VivaBlocks©) of the entire biopsy surface and applied a digital staining (DS) algorithm to pseudocolor images assigning purple to fluorescence and pink to reflectance signal resembling conventional HE staining.

### Histology

To corroborate our noninvasive imaging finding, we also collected 8-mm punch biopsies for conventional hematoxylin and eosin (HE) histology and Warthin-Starry (WS) stain, an argyrophilic silver nitrate stain for selective visualization of melanin [23]. Biopsies were embedded in optimal cutting temperature medium (Tissue TEK OCT) before sectioning on a microtome‐cryostat (Leica CM 3050S) at a thickness of 10 µm and examination under 40 × magnification using a slide scanner (MoticEasyScan Pro device, MoticEurope, Barcelona, Spain) and proprietary software suite.

## Results

Both clinically and histologically, the difference in pigmentation of the three skin types was visible and corresponded to the results of the objective melanin index measurements (MI range 0–100), with an MI of > 98 for dark-, 58.7 for medium-, and 24.5 for light-pigmented skin. Using three noninvasive imaging techniques, dermoscopy, OCT, and LC-OCT, and three tissue stains, HE, WS, and AO, a relationship between melanin density, clinically visible skin surface changes, and subclinical PSL-tissue interactions could be confirmed.

### Noninvasive visualization of PSL-tissue interactions

**Fig. 1:**
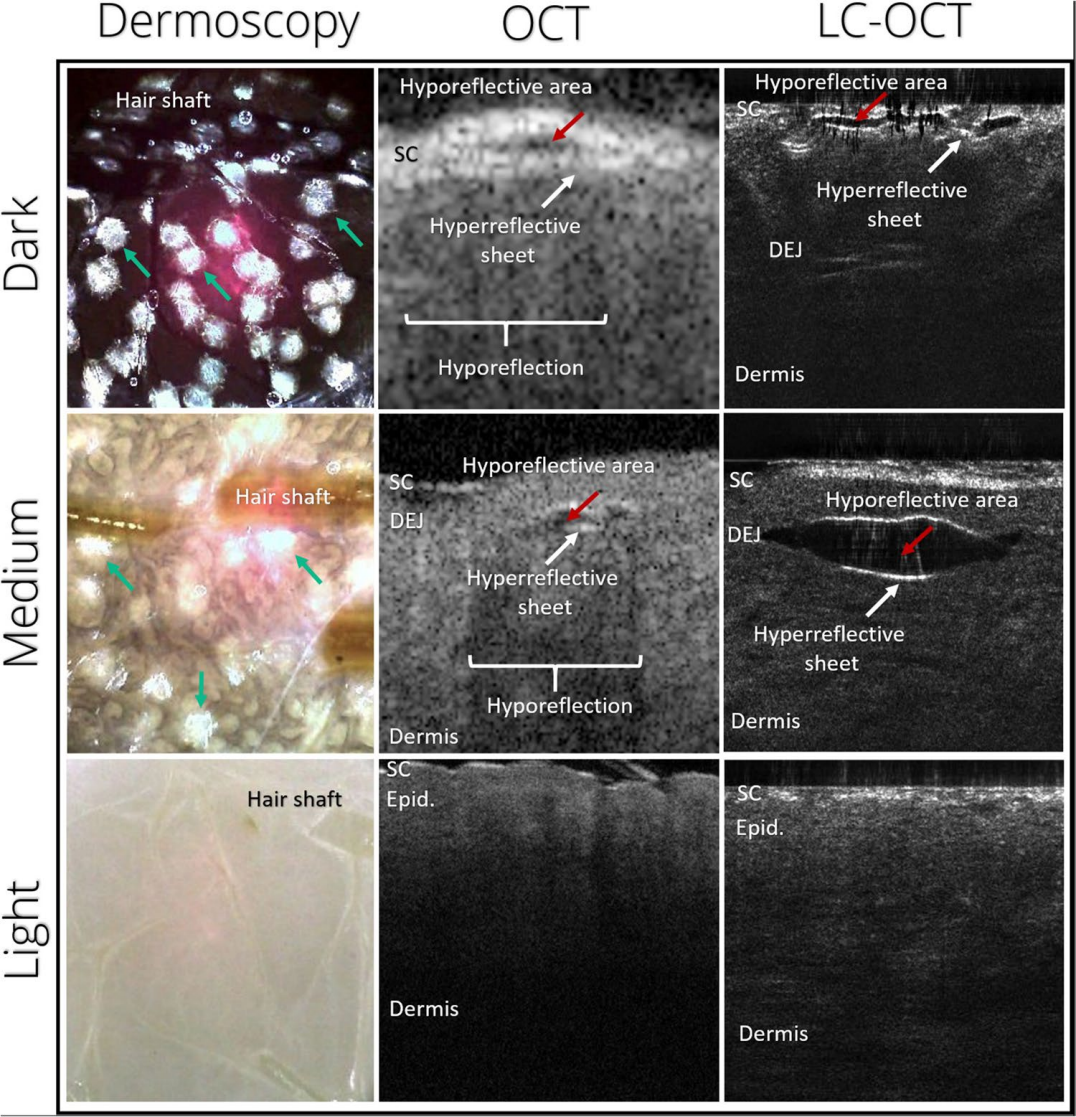
Visualization of tissue interactions induced by a 755-nm picosecond Alexandrite laser with a fractional optic using noninvasive optical imaging. Differences in location and density of intraepidermal vacuoles depending on melanin index. Dermoscopy shows focal whitening (green arrow) on the surface of the dark and medium-pigmented skin. Co-localized OCT and LC-OCT images show vacuoles located in the upper epidermis of the dark-, and the lower epidermis of the medium-pigmented skin, characterized by a hyperreflective outer shell (white arrow) encapsulating a hyporeflective cavity (red arrow). In OCT, focal shadowing is seen beneath vacuoles in dark and medium-pigmented skin. SC, stratum corneum; DEJ, dermo-epidermal junction; OCT, optical coherence tomography; LC-OCT, line-field confocal optical coherence tomography

Dermoscopy, LC-OCT, and OCT were capable of identifying morphological changes in PSL-treated porcine skin. Figure 1 shows representative images of immediate PSL-tissue interactions, including dermoscopic images of clinically visible surface changes and vacuoles in LC-OCT and OCT images.

**Fig. 2:**
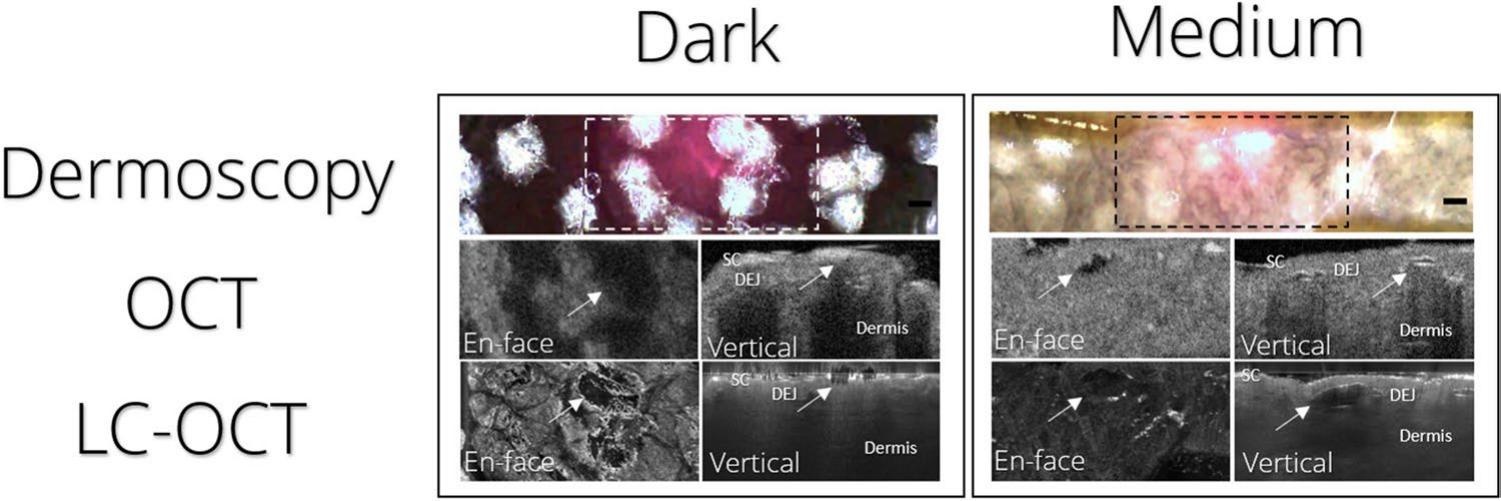
Clinical and subclinical skin changes induced by exposure to 755-nm picosecond-domain Alexandrite laser with a fractional optic. Clinically visible surface whitening visualized using dermoscopy (dashed outline) can be correlated to subclinical changes including vacuoles (white arrow) in OCT and LC-OCT in both dark- and medium-pigmented ex vivo porcine skin. SC, stratum corneum; DEJ, dermo-epidermal junction; LC-OCT, line-field confocal optical coherence tomography

In *dark-pigmented* skin, dermoscopy showed focal skin surface whitening with fading edges as well as depigmentation of hair shafts matching the grid-like pattern of the diffractive lens array (Fig. 2). In LC-OCT images, vacuoles appeared predominantly in the upper epidermis (Supplementary Fig. 1). In both cross-sectional and en face view, OCT visualized areas of PSL-exposed dark-pigmented skin with confluent, homogeneous shadowing with interspersed bright elements corresponding to vacuoles (Supplementary Fig. 2), visible at high magnification as hyporeflective space with a hyperreflective sheathing.

Dermoscopy of *medium-pigmented* skin showed a similar pattern as seen in dark skin, with focal whitening in a grid-like pattern. Medium-pigmented skin, in contrast, showed its highest density of vacuoles in LC-OCT images in the lower epidermis, clustering along the dermal–epidermal junction (DEJ), including occasional vacuoles in the upper dermis. Vacuoles were characterized using LC-OCT, presenting as a spindle-shaped hyporeflective space with a thin, sharply demarcated, hyperreflective lining surrounded by intact epidermis.

In OCT, the PSL-processed area could easily be delineated, with focal pinpoint shadowing visible below strongly scattering/hyperreflective elements in cross-sectional scans, corresponding to vacuoles (see Figs. 1 and 2). The less homogeneous, more dispersed pattern allowed for easy identification of individual vacuoles compared to dark-pigmented skin. The markedly decreased scatter response beneath vacuoles, visible as focal shadowing at depths corresponding to the location of vacuoles in LC-OCT.

As a consequence of these vacuoles and shadowing in dark- and medium-pigmented skin, the visibility of the dermal compartment deteriorated in OCT and LC-OCT images due to the decline in reflectance signal from PSL-treated areas.

In *light-pigmented* skin, dermoscopy showed no signs of skin surface changes. Further, the epidermal architecture was intact, with no clinically visible skin surface changes or any indications of vacuoles in LC-OCT (Fig. 1). In contrast to dark- and medium-pigmented skin, the dermal visibility mainly remained unaffected, with only a faint decrease in scatter response compared to untreated surrounding tissue in OCT.

Supplementary Fig. 3 shows examples of commonly found LC-OCT image artifacts in PSL-processed skin, including the decreased visibility of the dermis due to PSL-induced change in the refractive index in medium- and dark-pigmented skin. This loss of signal was also seen in OCT images, particularly pronounced in dark skin (Supplementary Fig. 2).

Untreated skin showed no visible difference other than pigmentation in dermoscopy images. OCT visualized a similar skin architecture in all skin types with poor contrast between the epidermis and dermis. LC-OCT showed a well-defined DEJ in dark- and medium-pigmented skin and a correlation between epidermal brightness and melanin density.

### Conventionally and digitally stained histology of PSL-tissue interactions

**Fig. 3:**
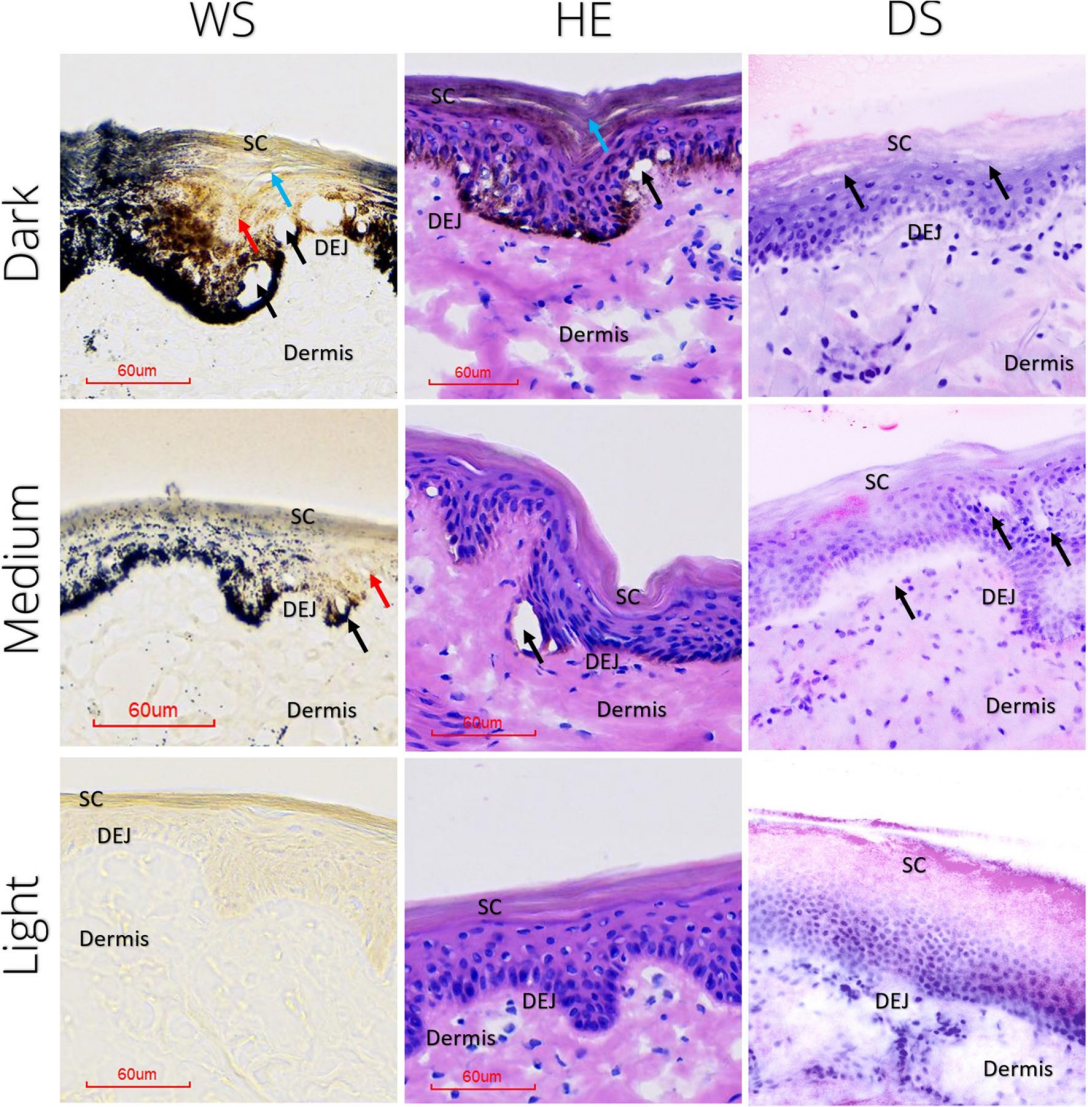
Subclinical 755-nm picosecond Alexandrite laser (PSL) effects in ex vivo porcine skin visualized using histological and digital staining. After treatment with a 755-nm PSL with a fractional optic, cryosections stained with Warthin-Starry (WS) and hematoxylin and eosin (HE) showed melanin-dependent micromorphologic changes. In dark- and medium-pigmented skin, vacuoles (black arrow) accompanied by areas void of WS staining in the epidermis (red arrow) can be observed. While intact in medium- and light-pigmented skin, the uppermost layers of the epidermis and stratum corneum of dark skin samples show localized architectural disarray (blue arrow). In the light-pigmented porcine skin, no signs of PSL-tissue interactions were visible. The digitally stained (DS) confocal microscopy images of bisected biopsies show morphological changes corresponding to conventional histology. PSL, picosecond laser; SC, stratum corneum; DEJ, dermo-epidermal junction; HE, hematoxylin and eosin; WS, Warthin-Starry; DS, digitally stained

In HE histology, and WS histology and DS-EVCM images, identification of vacuoles and associated PSL-tissue interactions was feasible. Figure 3 shows representative images of PSL-exposed dark-, medium-, and light-pigmented skin.

PSL-exposed *dark skin* presented with vacuoles in HE-stained sections and with a triad of (i) vacuoles accompanied by (ii) focal melanin fading visible as areas void of WS staining and (iii) a stratum corneum with signs of architectural disarray in WS-stained sections. Similarly, we observed vacuoles in biopsies scanned with EVCM located in the epidermis (Fig. 3).

PSL-exposed *medium-pigmented* skin also presented with vacuoles and melanin fading, but the stratum corneum appeared preserved. Moreover, the areas void of staining in WS sections appeared to be located more superficially in dark skin but span the entire epidermis in medium-pigmented skin samples. In HE-stained sections, melanin fading was more subtle than in WS and primarily visible at the basal membrane for dark- and medium-pigmented skin. Digitally stained EVCM was also able to identify vacuoles which, in line with our histology findings, appeared to be located deeper than in dark-pigmented skin, at the DEJ or just beneath (Fig. 3).

*Light-pigmented* skin showed no signs of vacuoles or other traces of PSL-tissue interactions, presenting with intact epidermal layers, no discernible melanin fading, and absence of characteristic vacuoles. Equally, the assessment of digitally stained EVCM images of PSL-exposed light-pigmented skin provided no noteworthy findings.

In both conventional histology and digitally stained EVCM images, artifacts were identified that complicated the description of PSL-tissue interactions. While cryosectioning introduced tissue tears that closely resemble vacuoles, improper mounting showed uneven tissue compression leading to incomplete visualization of the epidermis and the appearance of artificial gaps (see Supplementary Fig. 3).

In conventional HE histology of untreated *dark-pigmented* skin, we observed pigmentation along basal membrane corresponding to a high number and size of melanosomes. Due to the high density in melanin, WS staining colored the entire epidermis uniformly black.

In sections of *medium-pigmented* control skin, we found morphological changes similar to but more subdued than those in dark skin. While melanosomes were only faintly visible in HE-stained sections, WS-staining accentuated their visibility along the DEJ and demonstrated the high melanin density throughout the epidermis and stratum corneum.

Untreated *light-pigmented* skin was similar to medium- and dark-pigmented skin in appearance in HE-stained sections. In WS-stained sections, however, skin presented void of stain corresponding to its low melanin density. Untreated skin showed subtle differences in HE-stained sections. EVCM images did not show any pigment-specific differences (Fig. 3).

## Discussion

By combining three noninvasive and three biopsy-based visualization approaches, this study explored the spatial localization of vacuoles and the spectrum of accompanying tissue alterations in response to PSL treatment in relation to the cutaneous melanin density. The results of our investigation are twofold; first, melanin density influences the clinical and subclinical manifestation of PSL-tissue interactions; second, the complementary use of histological and optical imaging techniques can counterbalance technique-specific limitations, permitting a comprehensive visualization of PSL-tissue interactions.

Considering the human spectrum of cutaneous melanin density, knowledge of the different melanin-dependent PSL-tissue interactions may help refine the existing therapeutic PSL approaches. Our results confirmed previous findings on the spatial localization of vacuoles in relation to melanin density and suggested that the presence of melanin in superficial layers leads to superficially located vacuoles and acts as a protective shield preventing collateral PSL-induced damage in underlying layers. This theory is further supported by the confluent shadowing seen in OCT images of dark-pigmented skin.

As previously described, the fluence pattern of fractionated PSL treatment consists of 70% energy distributed over 10% of the treatment area, corresponding to the occurrence of vacuoles, and the remaining 30% between these fluence peaks, presumably leading to a subclinical change in scatter response in skin with high melanin density [12, 24, 25].

While this superficial epidermal absorption in darker skin types may prevent dermal hemorrhaging, [12] targeted disruption of the uppermost layer may prove beneficial in a drug delivery context. In a recent in vitro drug delivery study, the use of a picosecond-domain Nd:YAG laser permitted enhanced permeation of topically applied peptides [5]. In both medium- and dark-pigmented porcine skin, three passes resulted in clearly visible changes in the stratum corneum and epidermis indicative of a targeted disruption of the skin barrier function that may explain the mechanism behind non-ablative PSL drug delivery.

In a clinical context, melanin-dependent skin barrier disruption using PSL may be of particular interest in the delivery of topically applied drugs in the treatment of primary hyperpigmentation disorders such as melasma as well as secondary hyperpigmentation such as post-inflammatory hyperpigmentation [26].

Noninvasive imaging has a variety of advantages, including the ability to correlate clinically visible skin surface changes to subclinical changes, aiding in the spatio-temporal assessment of PSL-tissue interactions. LC-OCT, in particular, was able to visualize vacuoles and provide their location, whereas the benefit of OCT was in the identification and delineation of PSL-treated areas. Given the cellular resolution of LC-OCT, large field of view of OCT, and easy co-localization of PSL-tissue interactions, their complimentary use can facilitate a comprehensive bedside assessment.

A trade-off to noninvasive assessment is the compromised visibility in PSL-treated skin, with shadowing preventing a complete assessment of the dermis. Further, the insufficient optical resolution of OCT impaired visualization of vacuoles, whereas the small field of view of the LC-OCT limited the comparison of PSL-treated and adjacent untreated tissue. Lastly, the difference in laser wavelengths between OCT (1305 nm) and LC-OCT (800 nm) in relation to the absorption spectrum of melanin influences their ability to visualize cutaneous pigmentation and changes thereof.

The findings in WS-stained sections proved the added value of biopsy-based methods in the assessment of laser-tissue interactions by visualizing the bleaching of pigment visible as areas void of staining. Further, the use of EVCM allowed for complete and rapid visualization of the entire dermis. Biopsy-based methods were, however, prone to a variety of tissue processing artifacts. While lower in number and atypical in location, vacuoles similar to the ones seen in PSL-exposed skin were visible in histological sections and EVCM scans of control skin.

The melanin-dependent epidermal and dermal spatial distribution of vacuoles found in both LC-OCT and OCT was not consistently observed in our histological sections, where smaller vacuoles were found scattered in all melanin types in both PSL-treated and untreated samples. EVCM proved challenging as incomplete visualization of the epidermis and DEJ limited its capacity for detecting PSL-tissue interactions.

Uneven tissue compression was observed to result in image artifacts affecting the dermal papillae that could mimic the appearance of PSL-induced vacuoles. Previous freezing and storage of the skin, tissue processing artifacts, including disruption and folding of the upper skin layers due to sectioning and mounting of samples, complicated the interpretation and ultimately influenced the reliability of biopsy-based imaging. While EVCM was able to detect vacuoles, its inability to switch between en face and cross-sectional images and challenging tissue compression increase the risk of misinterpretation of artifacts and thereby limit its utility.

**Fig. 4:**
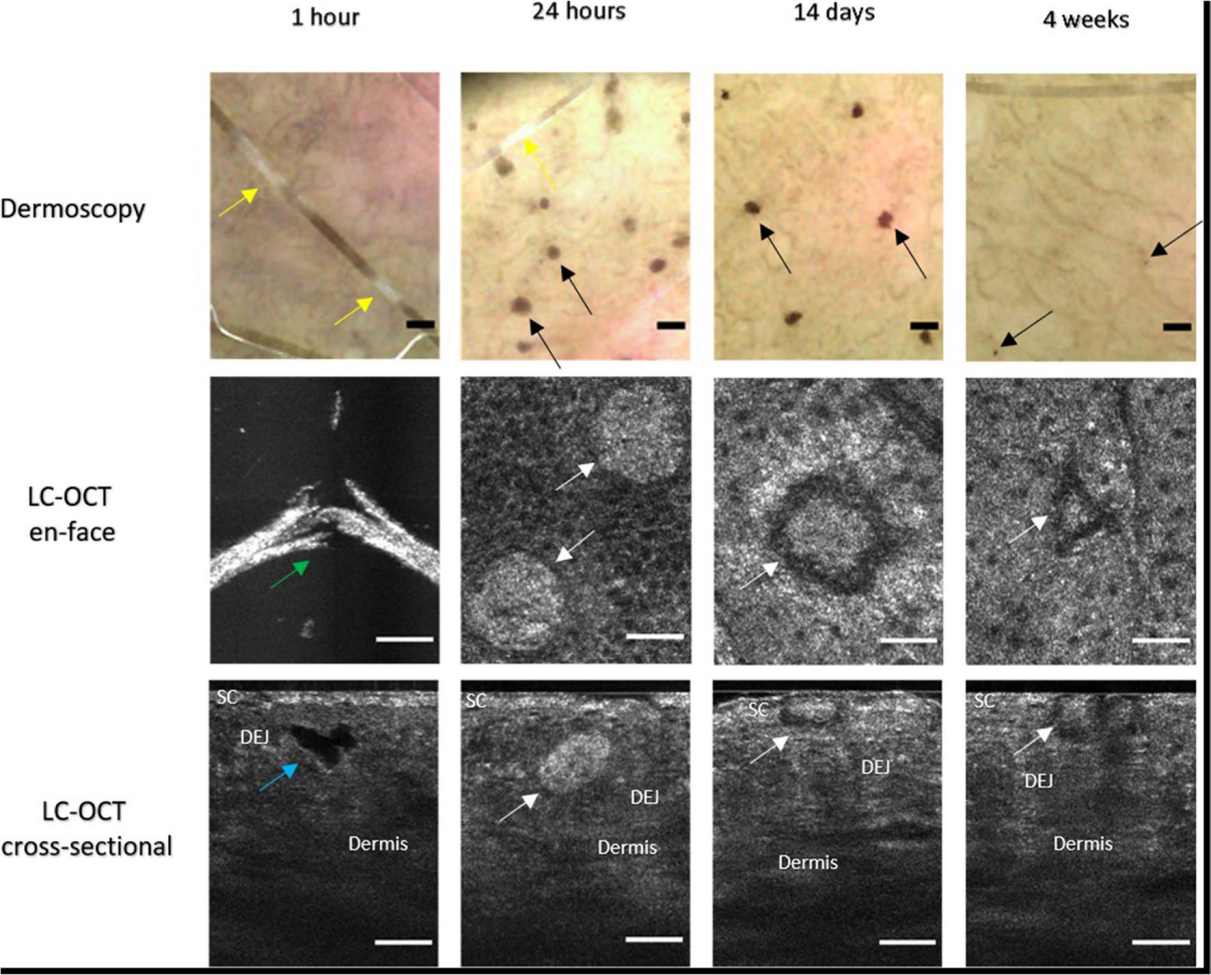
In vivo spatiotemporal LC-OCT detection of subclinical vacuoles in human volunteer with melanin index 27 after picosecond-domain 755-nm Alexandrite laser treatment with diffractive lens array. Dermoscopy shows hair follicle depigmentation (yellow arrow) immediately (1 h) after laser treatment. En face image shows fractured hair shaft (green arrow) and cross-sectional image an intra-epidermal vacuole (blue arrow). After 24 h, brown macules become visible in dermoscopy (black arrow) that diminish in size and number over time. In corresponding 24-h en face and cross-sectional images, hyperreflective oval structures, presumably former vacuoles filled with cellular debris (white arrows), become visible. After 14 days to 4 weeks, these histological elements gradually migrate through the epidermis to the skin surface. Scale bar in LC-OCT images, 100 µm. SC, stratum corneum; DEJ, dermo-epidermal junction; LC-OCT, line-field confocal optical coherence tomography

Strengths of this study include the use of six different assessments and skin with three objectively measured melanin densities. The major limitation of our results is the use of an ex vivo porcine model, which limits the application of our data to clinical practice. Building on the herein presented ex vivo findings, we piloted the feasibility of LC-OCT for the visualization of in vivo PSL-tissue interactions; their development in a skin type III, from vacuole formation to gradual migration toward the skin surface, can be observed in Fig. 4.

Larger in vivo investigations of the bedside use of noninvasive imaging to monitor PSL treatments in different skin types, as well as the development of quantitative approaches to the assessment PSL-tissue interactions, are warranted*.*

## Conclusion

Melanin-dependent variation in picosecond laser-tissue interactions in ex vivo porcine skin can be visualized using histology and optical imaging. Histology, in particular in combination with WS melanin staining, can visualize PSL-induced tissue alterations that may be of interest in the preclinical development of PSL devices. As noninvasive techniques appear less prone to critical artifacts that affect the visualization of epidermal vacuoles, the combined use of optical imaging and staining techniques can provide a complete picture of the spectrum of clinical and subclinical PSL-induced tissue interactions.

### Supplementary Information

Below is the link to the electronic supplementary material.

Supplementary file1 (JPG 150 KB)

Supplementary file2 (JPG 1623 KB)

Supplementary file3 (JPG 273 KB)

Supplementary file4 (DOCX 23 KB)

## Data Availability

Data is available upon reasonable request.
