# Supplementary figures and images for: Melanin-dependent tissue interactions induced by a 755-nm picosecond-domain laser: complementary visualization by optical imaging and histology
Source: Lasers Med Sci. 2023 Jul 14;38(1):160. doi: 10.1007/s10103-023-03811-4 (PMC10348935; doi:10.1007/s10103-023-03811-4)

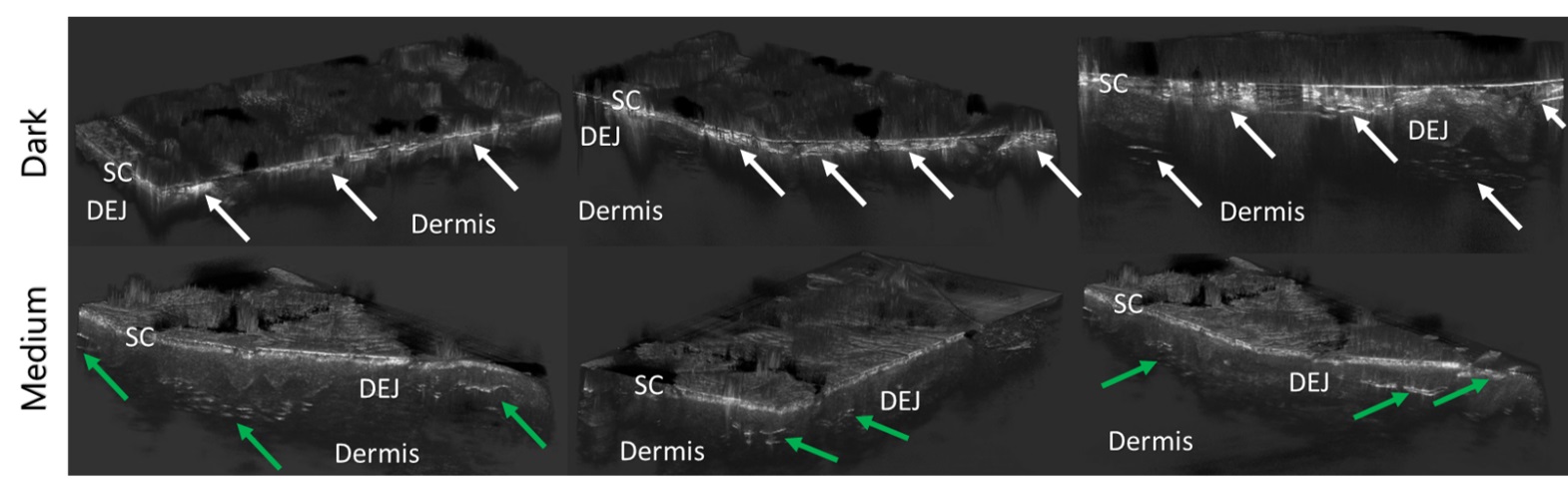

Supplement: Supplementary file 1 — Supplementary file1 (JPG 150 KB) [file 10103_2023_3811_MOESM1_ESM.jpg]

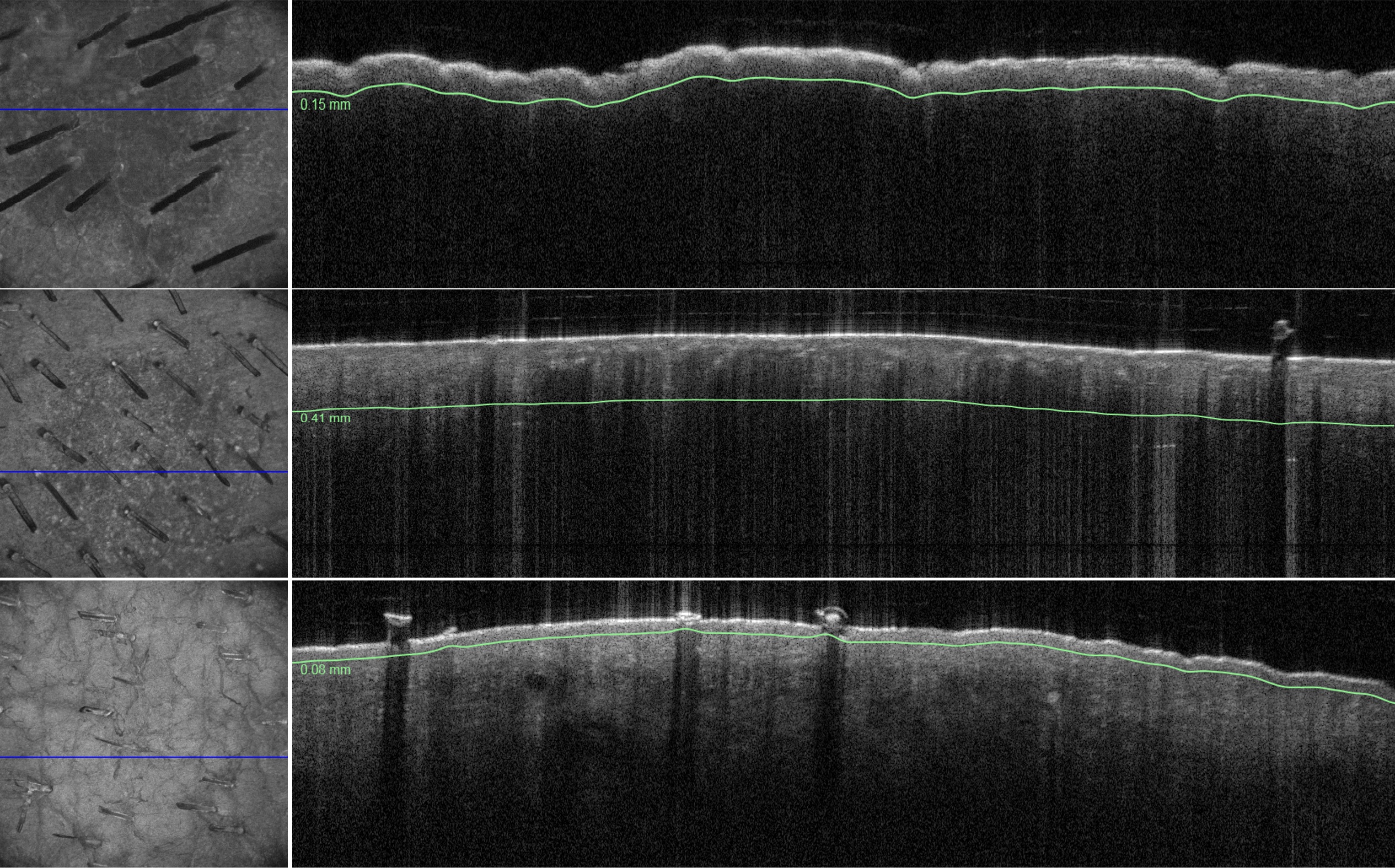

Supplement: Supplementary file 2 — Supplementary file2 (JPG 1623 KB) [file 10103_2023_3811_MOESM2_ESM.jpg]

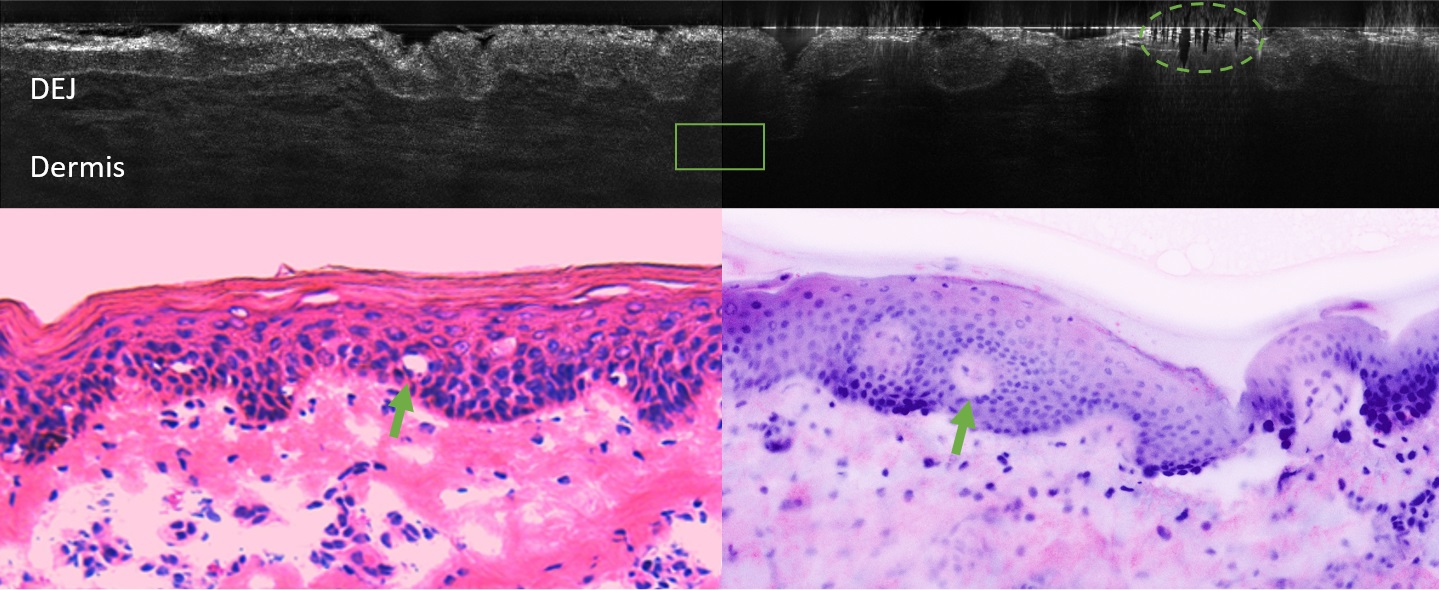

Supplement: Supplementary file 3 — Supplementary file3 (JPG 273 KB) [file 10103_2023_3811_MOESM3_ESM.jpg]
