# Supplementary material for: Melanin-dependent tissue interactions induced by a 755-nm picosecond-domain laser: complementary visualization by optical imaging and histology
Source: Lasers Med Sci. 2023 Jul 14;38(1):160. doi: 10.1007/s10103-023-03811-4 (PMC10348935; doi:10.1007/s10103-023-03811-4)
Supplement: Supplementary file 4 — Supplementary file4 (DOCX 23 KB) [file 10103_2023_3811_MOESM4_ESM.docx]

**Supplementary table 1:** Overview of 755 nm picosecond domain Alexandrite laser-induced tissue interactions reported in the literature

| Author (Year) | Laser settings | Treated tissue (MI) | Visualization method | Description (time point and morphology) |
| --- | --- | --- | --- | --- |
| Polnikorn N et al[26] (2020) | configured to deliver 0.71 J/cm2 with the 6 mm optic, 0.41 J/cm2 with the 8 mm optic, and 0.25 J/cm2 with the 10 mm optic. | *In vivo human* | Histology: HE | 24 hours: Areas of laser-induced optical breakdown (LIOB) from the absorption of high energy 755 nm light by melanin and were confined to the epidermis just below the granular layer without disruption to the basal layer at the dermal–epidermal (DE) junction |
| Choi MS et al[27] (2018) | 50 ps/2.8 mm/3.3 Jcm-2 | *in vivo* guinea pig (tattooed skin) | Histology: HE  Transmission electron microscopy | the 755 nm picosecond laser resulted in the least epidermal and dermal damages  EM: vesicular structures with an empty center and characteristic electron dense annular rim |
| Tanghetti E et al[25] (2017) | 0.25  J/ cm^2^, 0.4 J/ cm^2^, and 0.71 J/ cm^2^, | *in vivo* human (all skin types) | Histology: HE | Vacuoles were generally observed with the higher fluences. Rare areas of hemorrhage were seen only with the patient with an MI of 11. Intra-epidermal vacuoles were routinely observed in with the higher energies in patients with MI 18–24. In darker skin types, MI 30 and above, the vacuoles were seen with all the energies studied. |
| Tanghetti E[13] (2016) | Fractional Optic, fluence:  0.71 J/cm^2^,  0.40 J/ cm^2^  0.25 J/ cm^2^ | i*n vivo* human, (melanin index 10-64) | Histology: HE, Fontana Masson  in vivo RCM | No vacuoles found immediately after treatment in both histology and confocal imaging.  HE and Fontana Masson histology: At 5 days and 2 weeks MI 28-64, in darker skin types vacuoles found inferior to or within the SC, vacuoles only found at the DEJ with MI 10-12.  RCM: 24 hours after treatment, well-defined spherical vacuoles characterized by bright areas in the image were observed but only for subjects with MI > 15.  As the MI or fluence increased, the size and the density (number per unit area) of the vacuoles also increased. Vacuoles characterized as well-defined, spherical, intra-epidermal spaces. These vacuoles ranged in diameter from 25 to 70 μm and were located between 55 and 75 μm deep within the stratum spinosum. |
